# In vitro cytotoxicity of different dental resin-cements on human cell lines

**DOI:** 10.1007/s10856-020-06471-w

**Published:** 2021-01-20

**Authors:** Freya Diemer, Helmut Stark, Ernst-Heinrich Helfgen, Norbert Enkling, Rainer Probstmeier, Jochen Winter, Dominik Kraus

**Affiliations:** 1 Department of Oral Surgery, University of Bonn, Bonn, Germany; 2 Department of Prosthodontics, Preclinical Education and Dental Materials Science, University of Bonn, Bonn, Germany; 3 Department of Reconstructive Dentistry and Gerodontology, School of Dental Medicine, University of Bern, Bern, Switzerland; 4 Neuro- and Tumor Cell Biology Group, Department of Nuclear Medicine, University Hospital Bonn, Bonn, Germany; 5 Department of Periodontology, Operative and Preventive Dentistry, University of Bonn, Bonn, Germany

## Abstract

Adhesive resin-cements are increasingly used in modern dentistry. Nevertheless, released substances from resin materials have been shown to cause cellular toxic effects. Disc-shaped specimens from 12 different resin cements and one conventional zinc phosphate cement were prepared and used for direct stimulation of five different human cell lines via transwell cell culture system or in an indirect way using conditioned cell culture media. Cytotoxicity was determined using LDH and BCA assays. All tested cements led to a decrease of cell viability but to a distinct extent depending on cell type, luting material, and cytotoxicity assay. In general, cements exhibited a more pronounced cytotoxicity in direct stimulation experiments compared to stimulations using conditioned media. Interestingly, the conventional zinc phosphate cement showed the lowest impact on cell viability. On cellular level, highest cytotoxic effects were detected in osteoblastic cell lines. All resin cements reduced cell viability of human cells with significant differences depending on cell type and cement material. Especially, osteoblastic cells demonstrated a tremendous increase of cytotoxicity after cement exposure. Although the results of this in vitro study cannot be transferred directly to a clinical setting, it shows that eluted substances from resin cements may disturb osteoblastic homeostasis that in turn could lead to conditions favoring peri-implant bone destruction. Thus, the wide use of resin cements in every clinical situation should be scrutinized. A correct use with complete removal of all cement residues and a sufficient polymerization should be given the utmost attention in clinical usage.

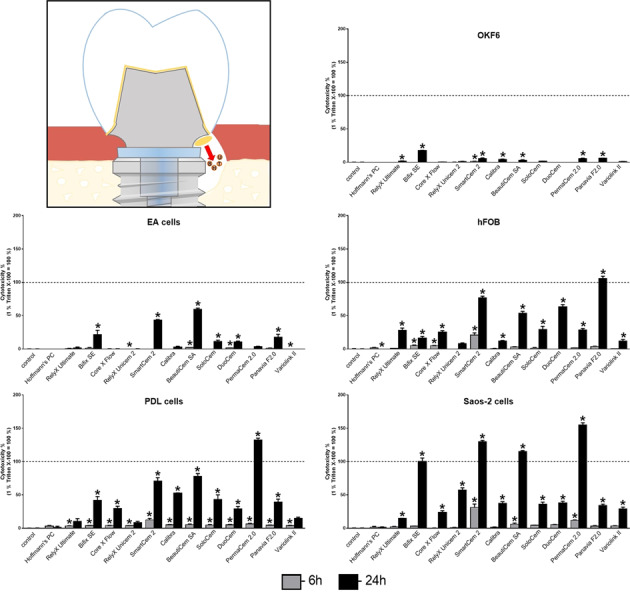

## Introduction

In reconstructive dentistry, cementation of indirect restorations is a crucial step in the treatment protocol and generally defines the finish of dental rehabilitation. While in the past, especially powder–liquid based cements such as zinc phosphate cements were used for fixation of restorations, in modern dentistry a numerous number of resin-based luting materials are utilized [1]. Due to their minimally invasive, preservative, and esthetic qualities, resin-based compounds have meanwhile become the most important material group in modern dentistry [2]. Compared to traditional cements, resin-based luting materials offer a high degree of color stability, adhesive linkage to dental hard tissue and other materials, low absorption of water with lower solubility at the same time, as well as better mechanical qualities [3–6]. Nevertheless, polymerization shrinkage can be mentioned as its major disadvantage [6]. Resin-based luting cements consist of an organic, polymerizable matrix, filler particles as well as different molecules, which evoke the polymerization reaction [3, 7, 8]. The organic matrix predominantly consists of cross-linking methacrylates, the so called “heavy” base monomer systems like 2,2-bis[4-(2-hydroxy-3-methacryloxypropoxy)phenyl]propane (BisGMA) and urethane dimethacrylate (UDMA) as well as different comonomers like triethyleneglycol dimethacrylate (TEGDMA) and 2-hydroxyethyl methacrylate (HEMA), which show a lower degree of viscosity [9–11]. The mechanical qualities of resin-based compounds, their viscosity, polymerization shrinkage, absorption of water, and their degree of conversion are predominantly determined by the composition of the monomers [12, 13]. Because of incomplete polymerization or inadequate qualities of the materials, elution of different ingredients as monomers can occur, which may lead to adverse reactions in surrounding tissues. Thus, negative biological consequences such as local or systemic toxicity, allergic and estrogenic effects are possible [10, 14–17].

The number and qualities of the differently dissolved components have already been proved and evaluated [10, 14, 18, 19]. Contact with alcoholic and organic solvents evokes higher release of the different ingredients compared to water. Furthermore, the cytotoxicity of the polymers widely depends on the type and quantity of the monomers [20–22]. Hydrophilic monomers such as HEMA and TEGDMA have been shown to dissolve in larger amounts compared to BisGMA and have the ability to diffuse through the dentine into the pulp chamber at concentrations of 1.5–8 mM [23, 24]. The highest levels of cytotoxicity on human oral fibroblasts derived from gingiva, dental pulp, and periodontal ligament (PDL) were determined in descending order for BisGMA, UDMA, TEGDMA, and HEMA, in which all acrylates depicted higher cytotoxicity than their corresponding methacrylates [21]. The process of elution in most cases, depending on the added solvent, ends a few days to weeks after the initial polymerization [10, 25].

A few number of recent in vitro studies are focused on the cellular and cytotoxic effects of various composite cements on oral fibroblasts such as dental pulp cells or permanent tumor cell lines [21, 22, 26]. In implant dentistry, peri-implant diseases are arising. Since composite cements are increasingly used in modern dentistry for the attachment of direct and indirect restorations, and especially in the context of implant prosthetics, it was found that residual cements play a significant role in the development of peri-implant inflammation (“cementitis”), the investigation of possible cytotoxic properties on bone cells is of great importance [3, 27, 28]. Thus, in a recent study, our working group evaluated the cytotoxic potential of different resin monomers on osteoblastic cell lines [29]. Already low, clinical relevant, monomer concentrations reduced cell viability of osteoblasts. Consequently, the objective of the present in vitro study was to investigate (i) the cytotoxicity of a substantial panel of different available dental resin-cements and a classical phosphate cement on a wide selection of human (oral) cell lines, and (ii) to compare the difference between to possible testing models (direct by transwell experiments vs. use of conditioned media). The data obtained from the study will extend our knowledge of the varying degrees of cytotoxic properties of the various cements and the different sensitivity of diverse cell types after challenging them with these cements.

## Materials and methods

### Production of disc-shaped cement specimens

Twelve commercially available (self-) adhesive resin-cements as well as one conventional zinc phosphate cement were tested in this study (Table 1). A total of 780 disc-shaped specimens with 5-mm diameter and 1-mm height (*n* = 60 per cement) were produced. At first, silicon sheets (Silflex Pink, Degudent) with 1-mm thickness were produced using a western blot gel casting apparatus (Mini-Protean handcast system, Biorad) with the 1-mm spacer plate. After the silicon was set, the sheets were removed from the casting frame, and holes with a diameter of 5 mm were punched out using a sterile biopsy punch. Decontamination of the silicon sheets was conducted by UV light radiation. In the next step, silicon sheets were placed on a glass plate and holes were filled with cement. Subsequently, a second glass plate was placed on top of the sheets, and all resin cements were directly light-cured from both sides, each 30 s. Light curing was performed using a poly-wave LED light-curing unit (Bluephase, Ivoclar Vivadent) with an irradiance of 1200 mW/cm^2^. This procedure assured a comparable homogeneous and smooth surface of all cement specimens without any more conditioning. For the zinc phosphate cement specimens, powder containing zinc oxide and magnesium oxide were mixed with liquid (o-phosphoric acid) at the ratio of 1.8-g powder: 1.0-g liquid. After all specimens were properly cured, cement discs were removed from the sheets and stored at 37 °C and 100% humidity in an incubator for 24 h.

**Table 1:** Listing of cements used for cytotoxic analysis

Cement	Manufacturer	Composition
BeautiCem SA	Shofu Dental GmbH	UDMA, HEMA, carboxylic acid monomer, phosphonate monomer
Bifix SE	VOCO GmbH	UDMA, BisEMA, glycerol dimethacrylate
Calibra Esthetic Resin Cement	Dentsply Sirona Deutschland GmbH	BisGMA, BisEMA, TEGDMA
Core × Flow	Dentsply Sirona Deutschland GmbH	UDMA, di- and trifunctional methacrylates
DuoCem	Coltène/Whaledent GmbH + Co. KG	BisGMA, TEGDMA
Hoffmann’s Phosphat Cement	Hoffmann Dental Manufaktur GmbH	Zinc oxide, magnesium oxide, o-phosphoric acid
PANAVIA F 2.0	Kuraray Europe GmbH	10-MDP, hydrophobic aromatic dimethacrylate, hydrophobic aliphatic dimethacrylate, hydrophilic aliphatic dimethacrylate
PermaCem 2.0	DMG Chemisch Pharmazeutische Fabrik GmbH	UDMA, TEGDMA, BisGMA
Rely × Ultimate	3M Deutschland GmbH	Mixture of mono-, di- and tri-glycerin-dimethacrylate-ester of phosphoric acid, TEGDMA, substituted dimethacrylate, 1,12-dodecaniol dimethacrylate
Rely × Unicem 2	3M Deutschland GmbH	Mixture of mono-, di- and tri-glycerin-dimethacrylate-ester of phosphoric acid, TEGDMA, substituted dimethacrylate, 1,12-dodecaniol dimethacrylate
SmartCem2	Dentsply Sirona Deutschland GmbH	UDMA, BisEMA, TEGDMA, HEMA, trimethylolpropane trimethacrylate, 3-(acryloyloxy)-2-hydroxypropyl methacrylate, dipentaerythritol pentaacrylate phosphate
SoloCem	Coltène/Whaledent GmbH + Co. KG	UDMA, TEGDMA, HEMA
Variolink Esthetic DC	Ivoclar Vivadent GmbH	UDMA, 1,10-decandiol dimethacrylate

In the column “Composition,” only (meth-) acrylic components without fillers, initiators, stabilizators, and pigments are specified for resin cements

*BisGMA* bisphenol A glycidilmethacrylate, *UDMA* urethane dimethacrylate, *BisEMA* bisphenol A ethoxylate dimethacrylate, *TEGDMA* triethylene glycol dimethacrylate, *HEMA* 2-hydroxyethyl methacrylate, *10-MDP* 10-methacryloyloxydecyl dihydrogen phosphate

### Cell culture

The following human cell lines were used in the experiments: OKF6/hTert-1 (OKF6; immortalized oral keratinocytes) from Rheinwald Laboratory (Harvard Medical School, USA), EA hy926 (EA cells; a permanent human umbilical vein cell line, which was established by fusing primary human umbilical vein cells with a thioguanine-resistant clone of A549 by exposure to polyethylene glycol) and hFOB 1.19 cells (hFOB; immortalized human fetal osteoblasts) purchased from ATCC (LGC Standards GmbH), osteoblast-like Saos-2 cells from Cell Line Service GmbH, and immortalized PDL cells kindly provided by Prof. Moritz Kebschull (Birmingham, UK). Cell culture conditions followed the recommendations from providers. All used cell culture media and supplements came from Thermo Fisher Scientific except stated otherwise. In brief, OKF6 cells were cultured in serum-free keratinocyte growth medium (KGM2; Promocell) supplemented with 1% antibiotic and antimycotic solution (AB), hFOBs were cultured in 1:1 mixture of Ham’s F12 Medium Dulbecco’s Modified Eagle’s Medium (DMEM) with 2.5-mM L-glutamine without phenol red and the addition of 0.3-mg/ml G418, 10% FCS, and 1% AB. Saos-2, EA, and PDL cells were cultured in DMEM containing 10% FCS and 1% AB. All cell lines were kept in an incubator at 37 °C (except hFOB at 34 °C) in a humidified atmosphere of 5% CO_2_ in air. Media were changed every 2–3 days. After reaching 80–90% confluency, cells were washed with Dulbecco’s phosphate-buffered saline (PBS) and detached from the culture vessels by a brief treatment with trypsin/EDTA (0.05/0.02%). Cell viability was evaluated using trypan blue solution (Sigma-Aldrich) followed by counting vital and dead cells. Contaminations with mycoplasma were routinely excluded by PCR analysis and DAPI staining.

### Cytotoxicity testing

Two different strategies were utilized to evaluate the cytotoxic potential of the test cement specimens. The first approach was to use the cement discs in a direct way using transwell cell culture plates. Therefore, cells were seeded in 24-well plates without inserts and cultured until they reached 90% confluency. Then, media were changed to 1-ml FCS-free media (OKF6 cells received fresh KGM2 with all supplements), cement discs (*n* = 6) were placed in cell culture inserts (one disc per insert; Falcon, Cell Culture Inserts, pore size 0.4 µm) and transferred to the 24-well plates with cells. Care was taken to ensure that cement specimens were totally covered with media after transferring the inserts to the 24-well plates. Wells without inserts and wells with inserts but without cement discs served as control. In addition, cells treated with 1% Triton X-100 (Sigma-Aldrich) served as positive control for the LDH assay. After an incubation period of 6 and 24 h at 37 °C, 100% humidity and 5% CO_2_ each 50-µl cell culture supernatant was removed from the wells to perform the LDH cytotoxicity assay. In addition, cell culture media left were removed from the wells after 24 h and cells were carefully washed twice with PBS. Then, cells were harvested for determination of total cellular protein content.

The second approach was to use conditioned media to incubate cells with eluted substances from cement specimens. Thus, cement discs (*n* = 6) were transferred to 24-well plates with one disc per well and 1-ml serum-free media or 1-ml KGM2 per well were added to the wells and discs. Subsequently, plates were placed in an incubator with 100% humidity at 37 °C for 24 h. During the incubation period, the plates were moved on an orbital shaker at low speed. Cell culture media without cement discs served as control whereas Triton X-100 (1%) treated cells were used for calculation in the LDH cytotoxicity assay. After 24 h, conditioned media were harvested and pooled for each cement group and directly used for further stimulation of the cells. Therefore, cells were seeded in 24-well plates and cultured until they reached 90% confluency. Then, media were changed to 1 ml conditioned media per well and each 50-µl supernatant was removed from the wells for LDH testing after an incubation period of 6 and 24 h. Finally, cells were harvested with cell lysis buffer after 24 h for total protein measurement.

### LDH assay

The Pierce LDH Cytotoxicity Assay kit (Thermo Fisher Scientific) was used in accordance to the manufacturer’s protocol. In brief, each 50-µl cell culture supernatant of stimulation experiments was transferred into 96-well plates. Then, 50 μl of LDH reaction mixture was added to the wells and incubated for 30 min in the dark at room temperature. The reaction was stopped by adding of 50-μl stop solution. Finally, the absorbance at 490 nm with correction wavelength at 680 nm was measured for every well, and cytotoxicity was calculated and normalized to positive control (Triton X-100 treatment was set to 100% cytotoxicity).

### BCA assay

In addition to the LDH assay, the measurement of total cellular protein level by the BCA assay was used as another method to determine cytotoxic effects of cement specimens after 24 h. Thus, after media were removed from wells, cells were carefully washed twice with cold PBS. Then, 100-µl cold lysis buffer (Cell Signaling Technologies) with proteinase inhibitors (Sigma-Aldrich) was added to cells, and plates were transferred on ice for 10 min. Subsequently, cell lysates were transferred to microtubes and centrifuged for 10 min at 14,000 × *g* in a cold microfuge. Resulting supernatants were directly used for the BCA assay (Pierce BCA Protein Assay Kit, Thermo Fisher Scientific) as recommended in the manufacturer’s protocol. Cell viability was calculated by dividing total protein content of control cells (=100% cell viability) through total protein content of cement-specimen-treated cells.

### Statistical analysis

**Fig. 1:**
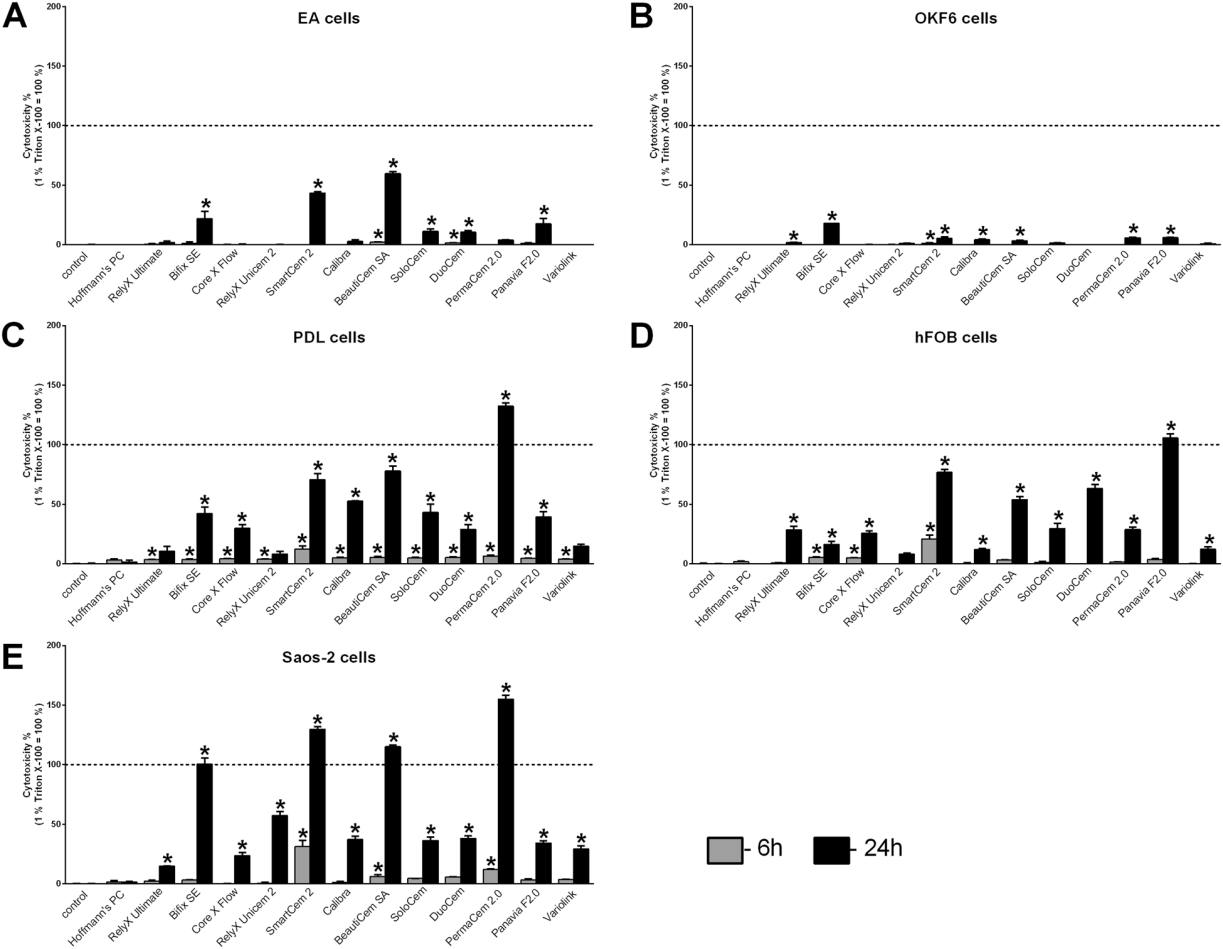
Cytotoxicity of luting cements after direct stimulation evaluated with the LDH assay. The cytotoxicity (LDH assay) of resin cements and zinc phosphate cement (Hoffmann’s PC) was evaluated in endothelial EA cells (**A**), immortalized oral epithelial OKF6 cells (**B**), immortalized PDL cells (**C**), immortalized human fetal osteoblastic hFOB cells (**D**), and Saos-2 cells (**E**) after direct stimulation using a transwell cell culture system for 6 (light gray bars) and 24 h (dark gray bars). Treatment with 1% Triton X-100 was used as positive control (=100% cytotoxicity). Mean ± SEM were calculated and one-way ANOVA and the post hoc Dunnett test were applied (*=*P* < 0.05)

**Fig. 2:**
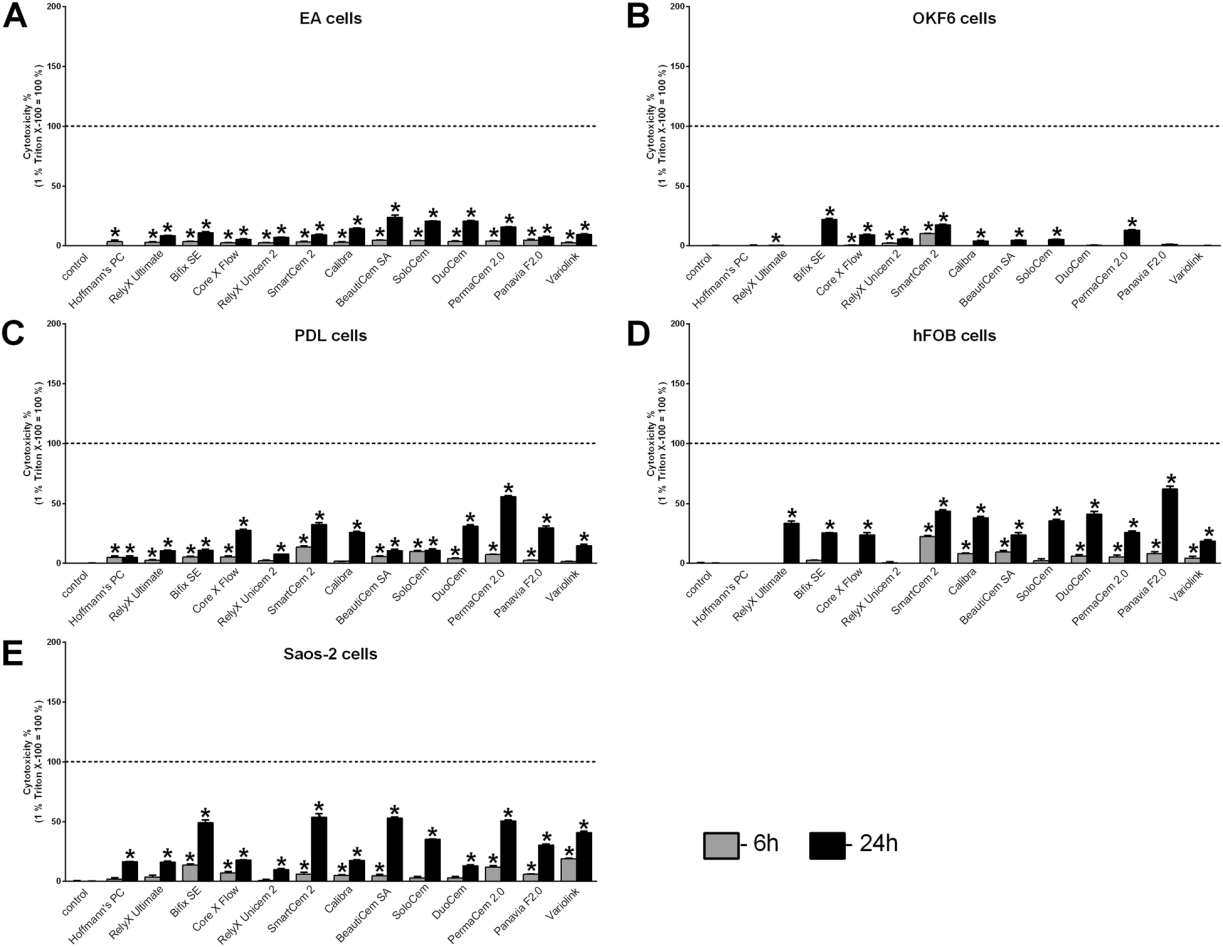
Cytotoxicity of luting cements after indirect stimulation evaluated with the LDH assay. The cytotoxicity (LDH assay) of resin cements and zinc phosphate cement (Hoffmann’s PC) was evaluated in endothelial EA cells (**A**), immortalized oral epithelial OKF6 cells (**B**), immortalized PDL cells (**C**), immortalized human fetal osteoblastic hFOB cells (**D**), and Saos-2 cells (**E**) after indirect stimulation using conditioned media for 6 (light gray bars) and 24 h (dark gray bars). Treatment with 1% Triton X-100 was used as positive control (=100% cytotoxicity). Mean ± SEM were calculated and one-way ANOVA and the post hoc Dunnett test were applied (*=*P* < 0.05)

**Fig. 3:**
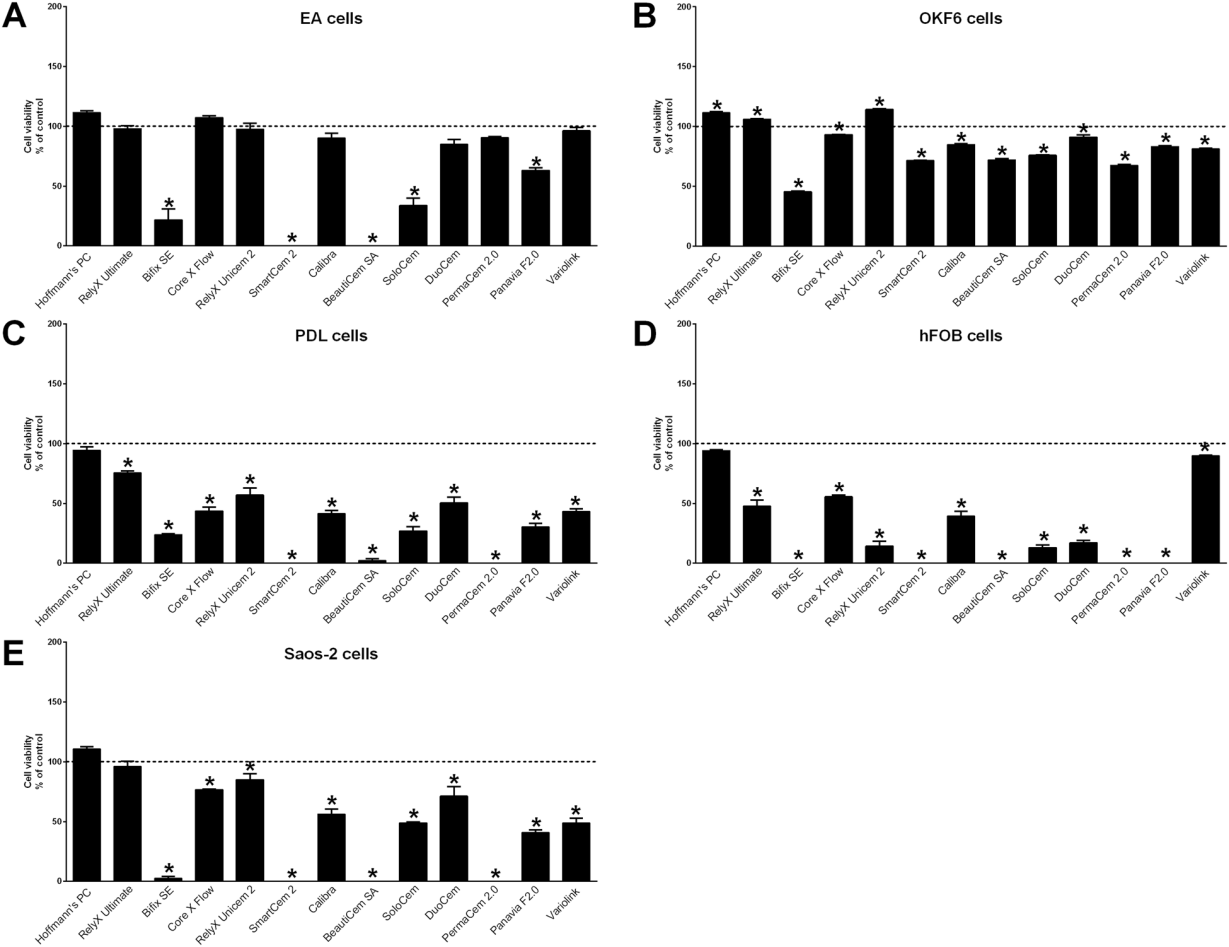
Cell viability after direct stimulation with luting cements evaluated with the BCA assay. Cell viability (BCA assay) of endothelial EA cells (**A**), immortalized oral epithelial OKF6 cells (**B**), immortalized PDL cells (**C**), immortalized human fetal osteoblastic hFOB cells (**D**), and Saos-2 cells (**E**) was evaluated after direct stimulation with resin cements and zinc phosphate cement (Hoffmann’s PC) using a transwell cell culture system for 24 h. Cells treated with cell culture inserts without cement specimen served as control (=100% cell viability). Mean ± SEM were calculated and one-way ANOVA and the post hoc Dunnett test were applied (*=*P* < 0.05)

**Fig. 4:**
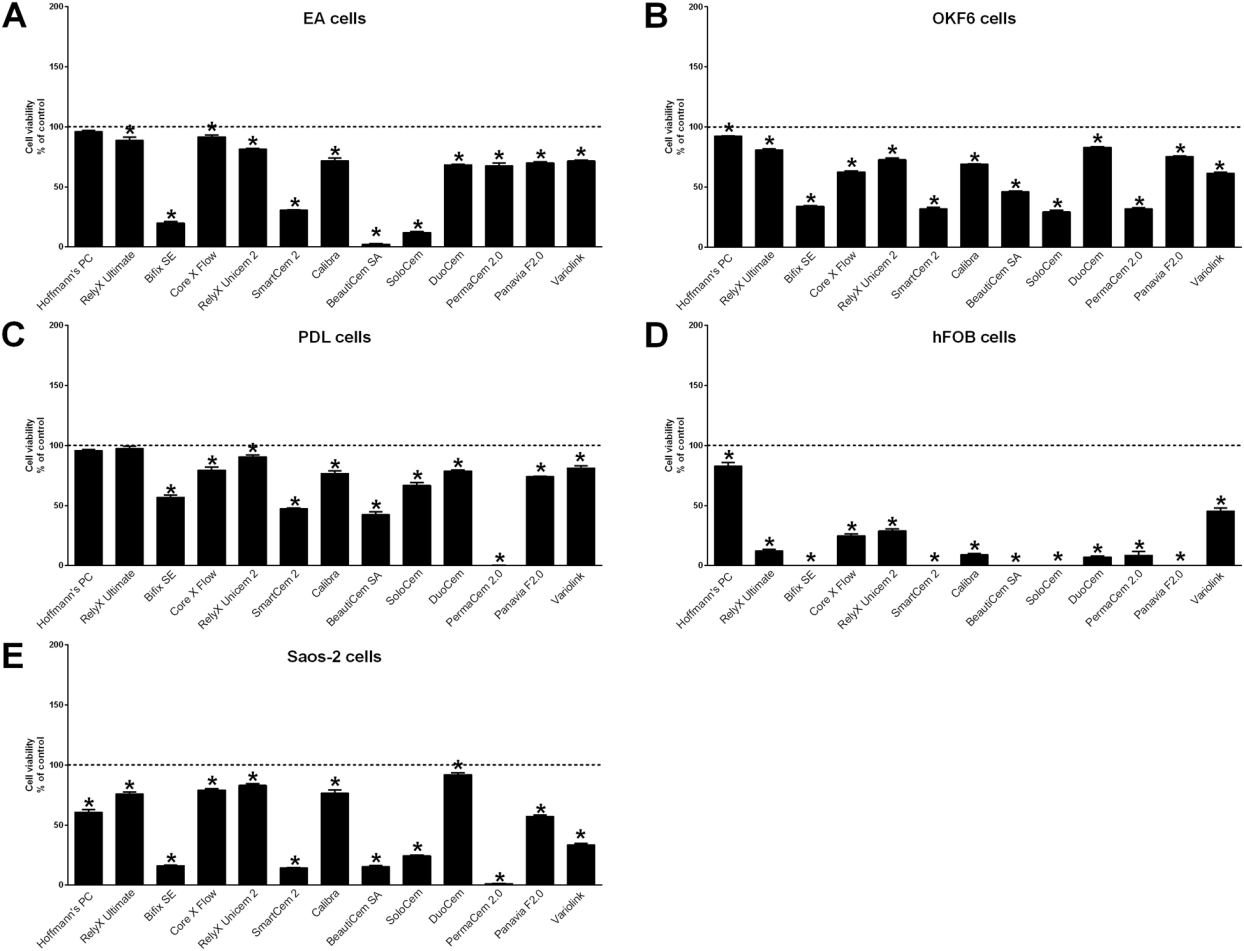
Cell viability after indirect stimulation with luting cements evaluated with the BCA assay. Cell viability (BCA assay) of endothelial EA cells (**A**), immortalized oral epithelial OKF6 cells (**B**), immortalized PDL cells (**C**), immortalized human fetal osteoblastic hFOB cells (**D**), and Saos-2 cells (**E**) was evaluated after direct stimulation with resin cements and zinc phosphate cement (Hoffmann’s PC) using conditioned media for 24 h. Cells stimulated with nonconditioned cell culture media served as control (=100% cell viability). Mean ± SEM were calculated and one-way ANOVA and the post hoc Dunnett test were applied (*=*P* < 0.05)

GraphPad Prism software, Version 6, (GraphPad Software) was used for statistical analysis. Mean ± standard error of the mean was calculated, and one-way ANOVA and the post hoc Tukey’s multiple comparison or Dunnett’s test were applied. *P* values < 0.05 were considered to be statistically significant. For better overview, only significant changes between cement groups and control are delineated in Figs. 1–4.

## Results

All used luting cements showed cytotoxic effects on cells been examined, whereby significant differences in cytotoxicity were detected between the different cements using both the LDH (Figs. 1 and 2) and the BCA assay (Figs. 3 and 4). Hoffmann’s Phosphat Cement showed lowest cytotoxic effects on all investigated cell lines. The strongest cytotoxic effect was demonstrated in transwell experiments for the cements SmartCem2, BeautiCem SA, PermaCem 2.0, and Bifix SE with values of 100% and more in Saos-2 cells after 24 h. In addition to the different degrees of cytotoxicity of the cements, the investigated cell lines showed varying degrees of sensitivity to the respective cements. For the tests with the LDH Assay Kit, this was in ascending order: OKF6 < EA < hFOB/PDL < Saos-2 and for testing with the BCA assay: OKF6 < EA < PDL < Saos-2 < hFOB. In addition, in all experiments and independently of the investigated cell and cement groups, the cements had a stronger cytotoxic effect by direct stimulation with the use of inserts (Figs. 1 and 3; values reached 100% and more; especially for mesenchymal cell lines) than by indirect stimulation with conditioned media (Figs. 2 and 4; a maximum value of 62% was detected for PANAVIA F 2.0 stimulation in hFOB cells after 24 h).

## Discussion

Adhesive luting cements are increasingly used in modern dentistry, as they are the means of choice for cementing ceramic restorations, have good mechanical properties, and are very easy to handle using automix application systems [30]. Nevertheless, various studies have investigated the cytotoxicity of resin-based dental materials on different (human) cell lines [31–37]. In addition, also cytotoxic effects of different resin luting cements on dental pulp and odontoblastic cells have been elucidated [38, 39]. The cytotoxicity of resin-based materials is primarily triggered by monomers released from the material [40–42]. The more unreacted substance the cured material contains, the higher is the toxic effect [42]. In most cases, the elution process is completed within the first few days or weeks after initial polymerization depending on the solvent [10, 25]. Especially the hydrophilic monomers HEMA and TEGDMA have been found to elute in higher amounts into aqueous extraction media as compared to BisGMA or UDMA [10, 23, 24, 43]. Altintas and Usumez evaluated the elution of a resin monomer from different luting cements after proper cementation of ceramic inlays in extracted tooth [13]. After immersion in a mix of ethanol/water (75/25 %), the release of TEGDMA was analyzed after different time points by HPLC. From all specimens, a TEGDMA release could be observed with highest total concentrations after 21 days ranging from 190 to 282 μM. Even 1 day after immersion, TEGDMA concentrations of 106–272 μM were detectable. Furthermore, Samanidou et al. evaluated the elution of different monomers from two composite resin-cements through dentine discs by HPLC analysis [8]. For Variolink cement, the release of TEGDMA, UDMA, and BisGMA could be confirmed whereas from the Multilink specimens only HEMA was detected in the elution medium. Overall, accumulated concentrations of TEGDMA, HEMA, BisGMA, and UDMA reached 0.16, 0.22, 0.14, and 0.05 mM, respectively. Although the results of both in vitro studies using organic solvents (ethanol/water and methanol) as elution medium cannot be directly transferred to the clinical situation in the oral cavity, the curing procedure of the composite resin-cements was performed under perfect laboratory conditions and may lead to higher degrees of conversion than in a real patient treatment. Thus, the detected monomer elution in both studies from the resin cements may be considered as maximum reachable concentrations. However, these values are definitely plausible in clinical situations where an inappropriate application of resin cement and additionally residual cement remnants occur.

In implant prosthetics, also adhesive luting cements are frequently used for fixation of supra-constructions. However, peri-implant diseases such as peri-implant mucositis and peri-implantitis represent significant biological complications of dental implants [44]. Thus, the prevalence of peri‐implantitis has been reported to range from 3 to 47% [45, 46]. In a recent study, ~1/3 of the patients and 1/5 of all implants have experienced peri-implantitis in a patient collective of an US dental school [47]. Here, the strongest putative risk factor was identified for an ill‐designed/ill‐fitting prosthesis (OR: 5.89, 95% CI: 1.65–21.11), which underlines the detrimental effects of persistent submucosal plaque retention at the implant–prosthesis interface. In addition, cement‐retained prosthesis (OR: 4.49, 95% CI: 2.12–9.50), presumably signifying leakage of cement deposits in the submucosal space, emerged as the second strongest risk factor. Thus, the complete removal of excess cement residues is a very important step after prosthetic placement to reduce the risk of peri-implant bone destruction [27, 48]. In this context, especially adhesive luting cements are difficult to remove due to their excellent bonding properties and their tooth-like color, which along with resin degradation and monomer release may lead to an increased peri-implant bone loss. In a recent study, our working group investigated the cytotoxic effects of common dental resin monomers on osteoblastic cell lines [29]. Indeed, low clinical relevant monomer concentrations led to a decrease in cell viability supporting the before-mentioned. Therefore, the objective of this new study was to go further on and to evaluate the cytotoxic potential in a wide panel of common dental resin-cements in different (oral) cell lines. Overall, all resin cements had an impact on cell viability in all tested cell systems. This is also reflected in a recent study of D’Alpino et al. [39]. They used self-adhesive resin-cements and tested their cytotoxicity after different curing protocols. In general, all tested specimen induced a significant decrease in cell viability of rat odontoblastic cells. Furthermore, if cements were only chemically cured, cytotoxicity was higher compared to specimens after light activation. This was also observed in a former study from Schmid-Schwap et al.: adhesive and self-adhesive resin-cements were significantly less cytotoxic when dual-cured compared to self-cured alone [32]. In our settings, all cement specimens were properly light-cured. Thus, without light activation, cytotoxicity could be expected to reach even higher values compared to our presented results. Interestingly, the cytotoxicity of zinc phosphate cement was lowest. This is in contrast to Schmid-Schwap et al. where the cytotoxicity ranged between ⁓13% (storage of cement specimen for 7 days in cell culture medium) and ⁓42% (fresh medium preparation) for zinc phosphate cement. This difference is may be due to (i) the use of different cell lines, (ii) other cell stimulation protocols, (iii) varying cell viability analysis, or (iv) different cement composition/supplier. Interestingly, although the same cement groups had a significant cytotoxic effect on all investigated cell groups, there were in part larger deviations in the sensitivity of the varying cell types after stimulation with the different cements. Thus, cell viability of both epithelial and endothelial cell line was generally reduced to a lesser extend compared to all three mesenchymal cells. Thus, the ranking of sensitivity of all tested cell lines against resin cements was in ascending order: OKF6 < EA < PDL < Saos-2 < hFOB cells. Here, especially in both osteoblastic cell lines, a tremendous increase of cytotoxicity could be proven which underlines possible detrimental effects of eluted substances from resin cements on osteoblastic homeostasis and therefore may lead to conditions favoring peri-implant diseases and bone destruction.

Our approach was to use a wide panel of commonly used resin cements for cytotoxic testing in a variety of different cell systems. Thus, the in vitro data should help the clinicians to choose the appropriate cement from a biological point of view besides a mechanical thinking. Nevertheless, the level of cytotoxicity of all tested cements in every used cell line showed a complete heterogeneous picture. There is no correlation between cytotoxicity and the adhesive characteristics of the cements on enamel/dentine (self-adhesion vs. additional use of a separate adhesive) or the composition of the luting specimens. In addition, the impact on cell viability for every cement depends on the tested cell system. As an example, PANAVIA F 2.0 showed an average cytotoxicity in almost all cell lines with the exception of hFOBs. Here, the strongest reduction of cell viability was detected. Taken all together, in our settings RelyX Unicem 2, RelyX Ultimate, and Variolink Esthetic demonstrated the lowest cytotoxic effects whereas SmartCem2, BeautiCem SA, PermaCem 2.0, and Bifix SE exhibited the strongest impact on cell viability.

## Conclusions

In conclusion, all resin cements reduced cell viability of human cells with significant differences depending on cell type and cement material. In our study, epithelial and endothelial cells showed a significantly lower sensitivity to cement exposure whereas mesenchymal cells and especially both osteoblastic cell lines demonstrated a tremendous increase of cytotoxicity. In our hands, the higher sensitivity of bone cells to resin-based materials should be taken into account in further dental materials biocompatibility studies. Although the results of this in vitro study cannot be transferred directly to a clinical setting, it shows that eluted substances from resin cements may disturb osteoblastic homeostasis that in turn could lead to conditions favoring peri-implant bone destruction. Despite of their good mechanical properties and the convenient handling, the wide use of composite resin-cements in every clinical situation should be scrutinized. In addition, a correct use with complete removal of all cement residues and a sufficient polymerization should be given the utmost attention in clinical usage.

## Supplementary information

Supplementary Table 1

Supplementary Table 2

Supplementary Table 3

Supplementary Table 4

Supplementary Table 5

Supplementary Table 6
