# Supplementary material for: In vitro cytotoxicity of different dental resin-cements on human cell lines
Source: J Mater Sci Mater Med. 2021 Jan 20;32(1):4. doi: 10.1007/s10856-020-06471-w (PMC7817560; doi:10.1007/s10856-020-06471-w)
Supplement: Supplementary file 1 — Supplementary Table 1 [file 10856_2020_6471_MOESM1_ESM.pdf]

Supplemental Table 1:

Multiple comparison analysis (one-way ANOVA with Tukey's post-hoc test) of cytotoxicity (LDH assay) in EA, OKF6, PDL, hFOB, and Saos-2 cells after direct stimulation with luting cements for 6 h.
* = $p < 0.05$, ** = $p < 0.01$, *** = $p < 0.001$.

**EA cells**	Hoffmann's PZ	RelyX Ultimate	Bifix SE	Core X Flow	RelyX Unicem 2	SmartCem2	Calibra	BeautiCem	SoloCem	DuoCem	Permazem	Panavia 2.0	Variolink Esthetic
Hoffmann's PZ								***		*			
RelyX Ultimate					***								**
Bifix SE					***	*	*						***
Core X Flow					**			**					
RelyX Unicem 2		***	***	**				***		***		***	
SmartCem2			*					***		**		*	
Calibra			*					***		**		*	
BeautiCem	***			**	***	***	***		**		***		***
SoloCem								**		*			
DuoCem	*				***	**	**		*		*		***
Permazem								***		*			
Panavia 2.0					***	*	*						***
Variolink Esthetic		**	***					***		***		***	

**OKF6 cells**	Hoffmann's PZ	RelyX Ultimate	Bifix SE	Core X Flow	RelyX Unicem 2	SmartCem2	Calibra	BeautiCem	SoloCem	DuoCem	Permazem	Panavia 2.0	Variolink Esthetic
Hoffmann's PZ						***							
RelyX Ultimate						***							
Bifix SE						***							
Core X Flow						***							
RelyX Unicem 2						***							
SmartCem2	***	***	***	***	***		***	***	***	***	***	***	***
Calibra						***							
BeautiCem						***							
SoloCem						***							
DuoCem						***							
Permazem						***							
Panavia 2.0						***							
Variolink Esthetic						***							

**PDL cells**	Hoffmann's PZ	RelyX Ultimate	Bifix SE	Core X Flow	RelyX Unicem 2	SmartCem2	Calibra	BeautiCem	SoloCem	DuoCem	Permazem	Panavia 2.0	Variolink Esthetic
Hoffmann's PZ						***							
RelyX Ultimate						***							
Bifix SE						***							
Core X Flow						***							
RelyX Unicem 2						***							
SmartCem2	***	***	***	***	***		***	***	***	***	***	***	***
Calibra						***							
BeautiCem						***							
SoloCem						***							
DuoCem						***							
Permazem						***							
Panavia 2.0						***							
Variolink Esthetic						***							

**hFOB cells**	Hoffmann's PZ	RelyX Ultimate	Bifix SE	Core X Flow	RelyX Unicem 2	SmartCem2	Calibra	BeautiCem	SoloCem	DuoCem	Permazem	Panavia 2.0	Variolink Esthetic
Hoffmann's PZ						***							
RelyX Ultimate						***							
Bifix SE					***	***				*			
Core X Flow					**	***				*			
RelyX Unicem 2			***	**		***						*	
SmartCem2	***	***	***	***	***		***	***	***	***	***	***	***
Calibra						***							
BeautiCem						***							
SoloCem						***							
DuoCem			*	*		***							
Permazem						***							
Panavia 2.0					*	***							
Variolink Esthetic						***							

**Saos-2 cells**	Hoffmann's PZ	RelyX Ultimate	Bifix SE	Core X Flow	RelyX Unicem 2	SmartCem2	Calibra	BeautiCem	SoloCem	DuoCem	Permazem	Panavia 2.0	Variolink Esthetic
Hoffmann's PZ						***					***		
RelyX Ultimate						***					**		
Bifix SE						***					**		
Core X Flow						***					***		
RelyX Unicem 2						***					***		
SmartCem2	***	***	***	***	***		***	***	***	***	***	***	***
Calibra						***					***		
BeautiCem						***							
SoloCem						***							
DuoCem						***							
Permazem	***	**	**	***	***	***	***					**	*
Panavia 2.0						***					**		
Variolink Esthetic						***					*		
