# Supplementary material for: In vitro cytotoxicity of different dental resin-cements on human cell lines
Source: J Mater Sci Mater Med. 2021 Jan 20;32(1):4. doi: 10.1007/s10856-020-06471-w (PMC7817560; doi:10.1007/s10856-020-06471-w)
Supplement: Supplementary file 2 — Supplementary Table 2 [file 10856_2020_6471_MOESM2_ESM.pdf]

Supplemental Table 2:

Multiple comparison analysis (one-way ANOVA with Tukey's post-hoc test) of cytotoxicity (LDH assay) in EA, OKF6, PDL, hFOB, and Saos-2 cells after direct stimulation with luting cements for 24 h.
* = $p < 0.05$, ** = $p < 0.01$, *** = $p < 0.001$.

**EA cells**	Hoffmann's PZ	RelyX Ultimate	Bifix SE	Core X Flow	RelyX Unicem 2	SmartCem2	Calibra	BeautiCem	SoloCem	DuoCem	Permazem	Panavia 2.0	Variolink Esthetic
Hoffmann's PZ			***			***		***	**	**		***	
RelyX Ultimate			***			***		***				***	
Bifix SE	***	***		***	***	***	***	***			***		***
Core X Flow			***			***		***				***	
RelyX Unicem 2			***			***		***				***	
SmartCem2	***	***	***	***	***		***	***	***	***	***	***	***
Calibra			***			***		***				**	
BeautiCem	***	***	***	***	***	***	***		***	***	***	***	***
SoloCem	**					***		***					
DuoCem	**					***		***					
Permazem			***			***		***				**	
Panavia 2.0	***	***		***	***	***	**	***			**		***
Variolink Esthetic			***			***		***				***	

**OKF6 cells**	Hoffmann's PZ	RelyX Ultimate	Bifix SE	Core X Flow	RelyX Unicem 2	SmartCem2	Calibra	BeautiCem	SoloCem	DuoCem	Permazem	Panavia 2.0	Variolink Esthetic
Hoffmann's PZ		*	***			***	***	***			***	***	
RelyX Ultimate	*		***			***	**			*	***	***	
Bifix SE	***	***		***	***	***	***	***	***	***	***	***	***
Core X Flow			***			***	***	***			***	***	
RelyX Unicem 2			***			***	***	*			***	***	
SmartCem2	***	***	***	***	***			*	***	***			***
Calibra	***	**	***	***	***				**	***			***
BeautiCem	***		***	***	*	*				***	**	**	*
SoloCem			***			***	**				***	***	
DuoCem		*	***			***	***	***			***	***	
Permazem	***	***	***	***	***			**	***	***			***
Panavia 2.0	***	***	***	***	***			**	***	***			***
Variolink Esthetic			***			***	***	*			***	***	

**PDL cells**	Hoffmann's PZ	RelyX Ultimate	Bifix SE	Core X Flow	RelyX Unicem 2	SmartCem2	Calibra	BeautiCem	SoloCem	DuoCem	Permazem	Panavia 2.0	Variolink Esthetic
Hoffmann's PZ			***	***		***	***	***	***	***	***	***	
RelyX Ultimate			***	*		***	***	***	***		***	***	
Bifix SE	***	***			***	***		***			***		***
Core X Flow	***	*			**	***	**	***			***		
RelyX Unicem 2			***	**		***	***	***	***	*	***	***	
SmartCem2	***	***	***	***	***				***	***	***	***	***
Calibra	***	***		**	***			***		**	***		***
BeautiCem	***	***	***	***	***		***		***	***	***	***	***
SoloCem	***	***			***	***		***			***		***
DuoCem	***				*	***	**	***			***		
Permazem	***	***	***	***	***	***	***	***	***	***		***	***
Panavia 2.0	***	***			***	***		***			***		**
Variolink Esthetic			***			***	***	***	***		***	**	

hFOB cells	Hoffmann's PZ	RelyX Ultimate	Bifix SE	Core X Flow	RelyX Unicem 2	SmartCem2	Calibra	BeautiCem	SoloCem	DuoCem	Permazem	Panavia 2.0	Variolink Esthetic
Hoffmann's PZ		***	***	***	***	***	***	***	***	***	***	***	***
RelyX Ultimate	***				***	***	**	***		***		***	**
Bifix SE	***					***		***	*	***	*	***	
Core X Flow	***				***	***	*	***		***		***	*
RelyX Unicem 2	***	***		***		***		***	***	***	***	***	
SmartCem2	***	***	***	***	***		***	***	***	*	***	***	***
Calibra	***	**		*		***		***	***	***	***	***	
BeautiCem	***	***	***	***	***	***	***		***		***	***	***
SoloCem	***		*		***	***	***	***		***		***	***
DuoCem	***	***	***	***	***	*	***		***		***	***	***
Permazem	***		*		***	***	***	***		***		***	***
Panavia 2.0	***	***	***	***	***	***	***	***	***	***	***		***
Variolink Esthetic	***	**		*		***		***	***	***	***	***	

Saos-2 cells	Hoffmann's PZ	RelyX Ultimate	Bifix SE	Core X Flow	RelyX Unicem 2	SmartCem2	Calibra	BeautiCem	SoloCem	DuoCem	Permazem	Panavia 2.0	Variolink Esthetic
Hoffmann's PZ		***	***	***	***	***	***	***	***	***	***	***	***
RelyX Ultimate	***		***	***	***	***	***	***	***	***	***	***	*
Bifix SE	***	***		***	***	***	***	*	***	***	***	***	***
Core X Flow	***	***	***		***	***	*	***	*	*	***		
RelyX Unicem 2	***	***	***	***		***	***	***	***	***	***	***	***
SmartCem2	***	***	***	***	***		***	*	***	***	***	***	***
Calibra	***	***	***	*	***	***		***			***		
BeautiCem	***	***	*	***	***	*	***		***	***	***	***	***
SoloCem	***	***	***	*	***	***		***			***		
DuoCem	***	***	***	*	***	***		***			***		
Permazem	***	***	***	***	***		***	***	***	***		***	***
Panavia 2.0	**	***	***		***	***		***			***		
Variolink Esthetic	***	*	***		***	***		***			***		
