# Supplementary material for: In vitro cytotoxicity of different dental resin-cements on human cell lines
Source: J Mater Sci Mater Med. 2021 Jan 20;32(1):4. doi: 10.1007/s10856-020-06471-w (PMC7817560; doi:10.1007/s10856-020-06471-w)
Supplement: Supplementary file 3 — Supplementary Table 3 [file 10856_2020_6471_MOESM3_ESM.pdf]

Supplemental Table 3:

Multiple comparison analysis (one-way ANOVA with Tukey's post-hoc test) of cytotoxicity (LDH assay) in EA, OKF6, PDL, hFOB, and Saos-2 cells after indirect stimulation with luting cements for 6 h.
* = p < 0.05, ** = p < 0.01, *** = p < 0.001.

**EA cells**	Hoffmann's PZ	RelyX Ultimate	Bifix SE	Core X Flow	RelyX Unicem 2	SmartCem2	Calibra	BeautiCem	SoloCem	DuoCem	Permazem	Panavia 2.0	Variolink Esthetic
Hoffmann's PZ													
RelyX Ultimate													
Bifix SE													
Core X Flow													
RelyX Unicem 2													
SmartCem2													
Calibra													
BeautiCem													
SoloCem													
DuoCem													
Permazem													
Panavia 2.0													
Variolink Esthetic													

**OKF6 cells**	Hoffmann's PZ	RelyX Ultimate	Bifix SE	Core X Flow	RelyX Unicem 2	SmartCem2	Calibra	BeautiCem	SoloCem	DuoCem	Permazem	Panavia 2.0	Variolink Esthetic
Hoffmann's PZ		***		***	***	***		*		**			
RelyX Ultimate	***		***		***	***	***	***	***	***	***	***	***
Bifix SE		***		***	***	***							
Core X Flow	***		***		***	***	***	***	***	***	***	***	***
RelyX Unicem 2	***	***	***	***		***	***	***	***	***	***	***	***
SmartCem2	***	***	***	***	***		***	***	***	***	***	***	***
Calibra		***		***	***	***							
BeautiCem	*	***		***	***	***			*				
SoloCem		***		***	***	***		*		*			
DuoCem	**	***		***	***	***			*				
Permazem		***		***	***	***							
Panavia 2.0		***		***	***	***							
Variolink Esthetic		***		***	***	***							

**PDL cells**	Hoffmann's PZ	RelyX Ultimate	Bifix SE	Core X Flow	RelyX Unicem 2	SmartCem2	Calibra	BeautiCem	SoloCem	DuoCem	Permazem	Panavia 2.0	Variolink Esthetic
Hoffmann's PZ					*	***	**		***				**
RelyX Ultimate				*		***		**	***		***		
Bifix SE					*	***	**		***			*	**
Core X Flow		*			**	***	***		***			*	***
RelyX Unicem 2	*		*	**		***		**	***		***		
SmartCem2	***	***	***	***	***		***	***	**	***	***	***	***
Calibra	**		**	***		***		***	***		***		
BeautiCem		**			**	***	***		***			**	***
SoloCem	***	***	***	***	***	**	***	***		***		***	***
DuoCem						***			***		**		
Permazem		***			***	***	***			**		***	***
Panavia 2.0			*	*		***		**	***		***		
Variolink Esthetic	**		**	***		***		***	***		***		

**hFOB cells**	Hoffmann's PZ	RelyX Ultimate	Bifix SE	Core X Flow	RelyX Unicem 2	SmartCem2	Calibra	BeautiCem	SoloCem	DuoCem	Permazem	Panavia 2.0	Variolink Esthetic
Hoffmann's PZ						***	***	***		***	**		
RelyX Ultimate						***	***	***		**	**	***	*
Bifix SE						***	*	**				*	
Core X Flow						***	***	***		**	**	***	*
RelyX Unicem 2						***	***	***		*		***	
SmartCem2	***	***	***	***	***		***	***	***	***	***	***	***
Calibra	***	***	*	***	***	***			*				
BeautiCem	***	***	**	***	***	***			***				
SoloCem						***	*	***				*	
DuoCem	***	**		**	*	***							
Permazem	**	**		**		***							
Panavia 2.0		***	*	***	***	***			*				
Variolink Esthetic		*		*		***							

**Saos-2 cells**	Hoffmann's PZ	RelyX Ultimate	Bifix SE	Core X Flow	RelyX Unicem 2	SmartCem2	Calibra	BeautiCem	SoloCem	DuoCem	Permazem	Panavia 2.0	Variolink Esthetic
Hoffmann's PZ			***	*							***		***
RelyX Ultimate			***								***		***
Bifix SE	***	***		**	***	***	***	***	***	***		***	*
Core X Flow	*		**		**								***
RelyX Unicem 2			***	**		*					***		***
SmartCem2			***		*						*		***
Calibra			***								**		***
BeautiCem			***								***		***
SoloCem			***								***		***
DuoCem			***								***		***
Permazem	***	***			***	*	**	***	***	***		**	***
Panavia 2.0			***								**		***
Variolink Esthetic	***	***	*	***	***	***	***	***	***	***	***	***	
