# Supplementary material for: In vitro cytotoxicity of different dental resin-cements on human cell lines
Source: J Mater Sci Mater Med. 2021 Jan 20;32(1):4. doi: 10.1007/s10856-020-06471-w (PMC7817560; doi:10.1007/s10856-020-06471-w)
Supplement: Supplementary file 4 — Supplementary Table 4 [file 10856_2020_6471_MOESM4_ESM.pdf]

Supplemental Table 4:

Multiple comparison analysis (one-way ANOVA with Tukey's post-hoc test) of cytotoxicity (LDH assay) in EA, OKF6, PDL, hFOB, and Saos-2 cells after indirect stimulation with luting cements for 24 h.
* = p < 0.05, ** = p < 0.01, *** = p < 0.001.

**EA cells**	Hoffmann's PZ	RelyX Ultimate	Bifix SE	Core X Flow	RelyX Unicem 2	SmartCem2	Calibra	BeautiCem	SoloCem	DuoCem	Permazem	Panavia 2.0	Variolink Esthetic
Hoffmann's PZ		***	***	***	***	***	***	***	***	***	***	***	***
RelyX Ultimate	***						***	***	***	***	***		
Bifix SE	***			***	*			***	***	***	***	*	
Core X Flow	***		***			*	***	***	***	***	***		*
RelyX Unicem 2	***		*				***	***	***	***	***		
SmartCem2	***			*			***	***	***	***	***		
Calibra	***	***		***	***	***		***	***	***		***	***
BeautiCem	***	***	***	***	***	***	***				***	***	***
SoloCem	***	***	***	***	***	***	***				**	***	***
DuoCem	***	***	***	***	***	***	***				**	***	***
Permazem	***	***	***	***	***	***		***	**	**		***	***
Panavia 2.0	***		*			***	***	***	***	***	***		
Variolink Esthetic	***			*			***	***	***	***	***		

**OKF6 cells**	Hoffmann's PZ	RelyX Ultimate	Bifix SE	Core X Flow	RelyX Unicem 2	SmartCem2	Calibra	BeautiCem	SoloCem	DuoCem	Permazem	Panavia 2.0	Variolink Esthetic
Hoffmann's PZ			***	***	***	***	***	***	***		***		
RelyX Ultimate			***	***	***	***	***	***	***		***		
Bifix SE	***	***		***	***	***	***	***	***	***	***	***	***
Core X Flow	***	***	***		***	***	***	***	***	***	***	***	***
RelyX Unicem 2	***	***	***	***		***				***	***	***	***
SmartCem2	***	***	***	***	***		***	***	***	***	***	***	***
Calibra	***	***	***	***		***				***	***	***	***
BeautiCem	***	***	***	***		***				***	***	***	***
SoloCem	***	***	***	***		***				***	***	***	***
DuoCem			***	***	***	***	***	***	***		***		
Permazem	***	***	***	***	***	***	***	***	***	***		***	***
Panavia 2.0			***	***	***	***	***	***	***		***		
Variolink Esthetic			***	***	***	***	***	***	***		***		

**PDL cells**	Hoffmann's PZ	RelyX Ultimate	Bifix SE	Core X Flow	RelyX Unicem 2	SmartCem2	Calibra	BeautiCem	SoloCem	DuoCem	Permazem	Panavia 2.0	Variolink Esthetic
Hoffmann's PZ		*	**	***		***	***	*	**	***	***	***	***
RelyX Ultimate	*			***		***	***			***	***	***	
Bifix SE	**			***		***	***			***	***	***	
Core X Flow	***	***	***		***	*		***	***		***		***
RelyX Unicem 2				***		***	***			***	***	***	***
SmartCem2	***	***	***	*	***		***	***	***		***		***
Calibra	***	***	***		***	***		***	***	*	***		***
BeautiCem	*			***		***	***			***	***	***	
SoloCem	**			***		***	***			***	***	***	
DuoCem	***	***	***		***		*	***	***		***		***
Permazem	***	***	***	***	***	***	***	***	***	***		***	***
Panavia 2.0	***	***	***		***			***	***		***		***
Variolink Esthetic	***			***	***	***	***			***	***	***	

**hFOB cells**	Hoffmann's PZ	RelyX Ultimate	Bifix SE	Core X Flow	RelyX Unicem 2	SmartCem2	Calibra	BeautiCem	SoloCem	DuoCem	Permazem	Panavia 2.0	Variolink Esthetic
Hoffmann's PZ		***	***	***	***	***	***	***	***	***	***	***	***
RelyX Ultimate	***		**	***	***	***		***		*	*	***	***
Bifix SE	***	**			***	***	***		***	***		***	
Core X Flow	***	***			***	***	***		***	***		***	
RelyX Unicem 2	***	***	***	***		***	***	***	***	***	***	***	***
SmartCem2	***	***	***	***	***			***	*		***	***	***
Calibra	***		***	***	***			***			***	***	***
BeautiCem	***	***			***	***	***		***	***		***	
SoloCem	***		***	***	***	*		***			***	***	***
DuoCem	***	*	***	***	***			***			***	***	***
Permazem	***	*			***	***	***		***	***		***	*
Panavia 2.0	***	***	***	***	***	***	***	***	***	***	***		***
Variolink Esthetic	***	***			***	***	***		***	***	*	***	

**Saos-2 cells**	Hoffmann's PZ	RelyX Ultimate	Bifix SE	Core X Flow	RelyX Unicem 2	SmartCem2	Calibra	BeautiCem	SoloCem	DuoCem	Permazem	Panavia 2.0	Variolink Esthetic
Hoffmann's PZ			***		*	***		***	***		***	***	***
RelyX Ultimate			***		*	***		***	***		***	***	***
Bifix SE	***	***		***	***		***		***	***		***	***
Core X Flow			***		**	***		***	***		***	***	***
RelyX Unicem 2	*	*	***	**		***	**	***	***		***	***	***
SmartCem2	***	***		***	***		***		***	***		***	***
Calibra			***		**	***		***	***		***	***	***
BeautiCem	***	***		***	***		***		***	***		***	***
SoloCem	***	***	***	***	***	***	***	***		***	***		
DuoCem			***			***		***	***		***	***	***
Permazem	***	***		***	***		***		***	***		***	***
Panavia 2.0	***	***	***	***	***	***	***	***		***	***		***
Variolink Esthetic	***	***	***	***	***	***	***	***		***	***	***	
