# Supplementary material for: In vitro cytotoxicity of different dental resin-cements on human cell lines
Source: J Mater Sci Mater Med. 2021 Jan 20;32(1):4. doi: 10.1007/s10856-020-06471-w (PMC7817560; doi:10.1007/s10856-020-06471-w)
Supplement: Supplementary file 5 — Supplementary Table 5 [file 10856_2020_6471_MOESM5_ESM.pdf]

## Supplemental Table 5:

Multiple comparison analysis (one-way ANOVA with Tukey's post-hoc test) of cytotoxicity (BCA assay) in EA, OKF6, PDL, hFOB, and Saos-2 cells after direct stimulation with luting cements for 24 h.
* = p < 0.05, ** = p < 0.01, *** = p < 0.001.

| **EA cells** | Hoffmann's PZ | RelyX Ultimate | Bifix SE | Core X Flow | RelyX Unicem 2 | SmartCem2 | Calibra | BeautiCem | SoloCem | DuoCem | Permazem | Panavia 2.0 | Variolink Esthetic |
|---|---|---|---|---|---|---|---|---|---|---|---|---|---|
| Hoffmann's PZ | | | *** | | | *** | * | *** | *** | ** | * | *** | |
| RelyX Ultimate | | | *** | | | *** | | *** | *** | | | *** | |
| Bifix SE | *** | *** | | *** | *** | * | *** | * | | *** | *** | *** | *** |
| Core X Flow | | | *** | | | *** | | *** | *** | ** | | *** | |
| RelyX Unicem 2 | | | *** | | | *** | | *** | *** | | | *** | |
| SmartCem2 | *** | *** | * | *** | *** | | *** | | *** | *** | *** | *** | *** |
| Calibra | * | | *** | | | *** | | *** | *** | | | *** | |
| BeautiCem | *** | *** | * | *** | *** | | *** | | *** | *** | *** | *** | *** |
| SoloCem | *** | *** | | *** | *** | *** | *** | *** | | *** | *** | *** | *** |
| DuoCem | *** | | *** | ** | | *** | | *** | *** | | | * | |
| Permazem | * | | *** | | | *** | | *** | *** | | | *** | |
| Panavia 2.0 | *** | *** | *** | *** | *** | *** | *** | *** | *** | * | *** | | *** |
| Variolink Esthetic | | | *** | | | *** | | *** | *** | | | *** | |

| **OKF6 cells** | Hoffmann's PZ | RelyX Ultimate | Bifix SE | Core X Flow | RelyX Unicem 2 | SmartCem2 | Calibra | BeautiCem | SoloCem | DuoCem | Permazem | Panavia 2.0 | Variolink Esthetic |
|---|---|---|---|---|---|---|---|---|---|---|---|---|---|
| Hoffmann's PZ | | ** | *** | *** | | *** | *** | *** | *** | *** | *** | *** | *** |
| RelyX Ultimate | ** | | *** | *** | *** | *** | *** | *** | *** | *** | *** | *** | *** |
| Bifix SE | *** | *** | | *** | *** | *** | *** | *** | *** | *** | *** | *** | *** |
| Core X Flow | *** | *** | *** | | *** | *** | *** | *** | *** | | *** | *** | *** |
| RelyX Unicem 2 | | *** | *** | *** | | *** | *** | *** | *** | *** | *** | *** | *** |
| SmartCem2 | *** | *** | *** | *** | *** | | *** | | * | *** | | *** | *** |
| Calibra | *** | *** | *** | *** | *** | *** | | *** | *** | *** | *** | | |
| BeautiCem | *** | *** | *** | *** | *** | | *** | | | *** | * | *** | *** |
| SoloCem | *** | *** | *** | *** | *** | * | *** | | | *** | *** | *** | ** |
| DuoCem | *** | *** | *** | | *** | *** | *** | *** | *** | | *** | *** | *** |
| Permazem | *** | *** | *** | *** | *** | | *** | * | *** | *** | | *** | *** |
| Panavia 2.0 | *** | *** | *** | *** | *** | *** | | *** | *** | *** | *** | | |
| Variolink Esthetic | *** | *** | *** | *** | *** | *** | | *** | ** | *** | *** | | |

| **PDL cells** | Hoffmann's PZ | RelyX Ultimate | Bifix SE | Core X Flow | RelyX Unicem 2 | SmartCem2 | Calibra | BeautiCem | SoloCem | DuoCem | Permazem | Panavia 2.0 | Variolink Esthetic |
|---|---|---|---|---|---|---|---|---|---|---|---|---|---|
| Hoffmann's PZ | | ** | *** | *** | *** | *** | *** | *** | *** | *** | *** | *** | *** |
| RelyX Ultimate | ** | | *** | *** | ** | *** | *** | *** | *** | *** | *** | *** | *** |
| Bifix SE | *** | *** | | *** | *** | *** | ** | *** | | *** | *** | | *** |
| Core X Flow | *** | *** | *** | | | *** | | *** | ** | | *** | | |
| RelyX Unicem 2 | *** | ** | *** | | | *** | * | *** | *** | | *** | *** | |
| SmartCem2 | *** | *** | *** | *** | *** | | *** | | *** | *** | | *** | *** |
| Calibra | *** | *** | ** | | * | *** | | *** | * | | *** | | |
| BeautiCem | *** | *** | *** | *** | *** | | *** | | *** | *** | | *** | *** |
| SoloCem | *** | *** | | ** | *** | *** | * | *** | | *** | *** | | * |
| DuoCem | *** | *** | *** | | | *** | | *** | *** | | *** | *** | |
| Permazem | *** | *** | *** | *** | *** | | *** | | *** | *** | | *** | *** |
| Panavia 2.0 | *** | *** | | | *** | *** | | *** | | *** | *** | | |
| Variolink Esthetic | *** | *** | *** | | | *** | | *** | * | | *** | | |

| hFOB cells | Hoffmann's PZ | RelyX Ultimate | Bifix SE | Core X Flow | RelyX Unicem 2 | SmartCem2 | Calibra | BeautiCem | SoloCem | DuoCem | Permazem | Panavia 2.0 | Variolink Esthetic |
|---|---|---|---|---|---|---|---|---|---|---|---|---|---|
| Hoffmann's PZ | | *** | *** | *** | *** | *** | *** | *** | *** | *** | *** | *** | |
| RelyX Ultimate | *** | | *** | | *** | *** | | *** | *** | *** | *** | *** | *** |
| Bifix SE | *** | *** | | *** | ** | | *** | | * | *** | | | *** |
| Core X Flow | *** | | *** | | *** | *** | *** | *** | *** | *** | *** | *** | *** |
| RelyX Unicem 2 | *** | *** | ** | *** | | ** | *** | ** | | | ** | ** | *** |
| SmartCem2 | *** | *** | | *** | ** | | *** | | * | *** | | | *** |
| Calibra | *** | | *** | *** | *** | *** | | *** | *** | *** | *** | *** | *** |
| BeautiCem | *** | *** | | *** | ** | | *** | | * | *** | | | *** |
| SoloCem | *** | *** | * | *** | | * | *** | * | | | * | * | *** |
| DuoCem | *** | *** | *** | *** | | *** | *** | *** | | | *** | *** | *** |
| Permazem | *** | *** | | *** | ** | | *** | | * | *** | | | *** |
| Panavia 2.0 | *** | *** | | *** | ** | | *** | | * | *** | | | *** |
| Variolink Esthetic | | *** | *** | *** | *** | *** | *** | *** | *** | *** | *** | *** | |

| Saos-2 cells | Hoffmann's PZ | RelyX Ultimate | Bifix SE | Core X Flow | RelyX Unicem 2 | SmartCem2 | Calibra | BeautiCem | SoloCem | DuoCem | Permazem | Panavia 2.0 | Variolink Esthetic |
|---|---|---|---|---|---|---|---|---|---|---|---|---|---|
| Hoffmann's PZ | | | *** | *** | *** | *** | *** | *** | *** | *** | *** | *** | *** |
| RelyX Ultimate | | | *** | * | | *** | *** | *** | *** | *** | *** | *** | *** |
| Bifix SE | *** | *** | | *** | *** | | *** | | *** | *** | | *** | *** |
| Core X Flow | *** | * | *** | | | *** | ** | *** | *** | | *** | *** | *** |
| RelyX Unicem 2 | *** | | *** | | | *** | *** | *** | *** | | *** | *** | *** |
| SmartCem2 | *** | *** | | *** | *** | | *** | | *** | *** | | *** | *** |
| Calibra | *** | *** | *** | ** | *** | *** | | *** | | | *** | | |
| BeautiCem | *** | *** | | *** | *** | | *** | | *** | *** | | *** | *** |
| SoloCem | *** | *** | *** | *** | *** | *** | | *** | | ** | *** | | |
| DuoCem | *** | *** | *** | | | *** | | *** | ** | | *** | *** | ** |
| Permazem | *** | *** | | *** | *** | | *** | | *** | *** | | *** | *** |
| Panavia 2.0 | *** | *** | *** | *** | *** | *** | | *** | | *** | *** | | |
| Variolink Esthetic | *** | *** | *** | *** | *** | *** | | *** | | ** | *** | | |
