# Supplementary material for: In vitro cytotoxicity of different dental resin-cements on human cell lines
Source: J Mater Sci Mater Med. 2021 Jan 20;32(1):4. doi: 10.1007/s10856-020-06471-w (PMC7817560; doi:10.1007/s10856-020-06471-w)
Supplement: Supplementary file 6 — Supplementary Table 6 [file 10856_2020_6471_MOESM6_ESM.pdf]

Supplemental Table 6:

Multiple comparison analysis (one-way ANOVA with Tukey's post-hoc test) of cytotoxicity (BCA assay) in EA, OKF6, PDL, hFOB, and Saos-2 cells after indirect stimulation with luting cements for 24 h.
* = $p < 0.05$, ** = $p < 0.01$, *** = $p < 0.001$.

**EA cells**	Hoffmann's PZ	RelyX Ultimate	Bifix SE	Core X Flow	RelyX Unicem 2	SmartCem2	Calibra	BeautiCem	SoloCem	DuoCem	Permazem	Panavia 2.0	Variolink Esthetic
Hoffmann's PZ		*	***		***	***	***	***	***	***	***	***	***
RelyX Ultimate	*		***		*	***	***	***	***	***	***	***	***
Bifix SE	***	***		***	***	***	***	***	**	***	***	***	***
Core X Flow			***		***	***	***	***	***	***	***	***	***
RelyX Unicem 2	***	*	***	***		***	***	***	***	***	***	***	***
SmartCem2	***	***	***	***	***		***	***	***	***	***	***	***
Calibra	***	***	***	***	***	***		***	***				
BeautiCem	***	***	***	***	***	***	***		***	***	***	***	***
SoloCem	***	***	**	***	***	***	***	***		***	***	***	***
DuoCem	***	***	***	***	***	***		***	***				
Permazem	***	***	***	***	***	***		***	***				
Panavia 2.0	***	***	***	***	***	***		***	***				
Variolink Esthetic	***	***	***	***	***	***		***	***				

**OKF6 cells**	Hoffmann's PZ	RelyX Ultimate	Bifix SE	Core X Flow	RelyX Unicem 2	SmartCem2	Calibra	BeautiCem	SoloCem	DuoCem	Permazem	Panavia 2.0	Variolink Esthetic
Hoffmann's PZ		***	***	***	***	***	***	***	***	***	***	***	***
RelyX Ultimate	***		***	***	***	***	***	***	***		***	**	***
Bifix SE	***	***		***	***		***	***	*	***		***	***
Core X Flow	***	***	***		***	***	***	***	***	***	***	***	
RelyX Unicem 2	***	***	***	***		***		***	***	***	***		***
SmartCem2	***	***		***	***		***	***		***		***	***
Calibra	***	***	***	***		***		***	***	***	***	***	***
BeautiCem	***	***	***	***	***	***	***		***	***	***	***	***
SoloCem	***	***	*	***	***		***	***		***		***	***
DuoCem	***		***	***	***	***	***	***	***		***	***	***
Permazem	***	***		***	***		***	***		***		***	***
Panavia 2.0	***	**	***	***		***	***	***	***	***	***		***
Variolink Esthetic	***	***	***		***	***	***	***	***	***	***	***	

**PDL cells**	Hoffmann's PZ	RelyX Ultimate	Bifix SE	Core X Flow	RelyX Unicem 2	SmartCem2	Calibra	BeautiCem	SoloCem	DuoCem	Permazem	Panavia 2.0	Variolink Esthetic
Hoffmann's PZ			***	***		***	***	***	***	***	***	***	***
RelyX Ultimate			***	***		***	***	***	***	***	***	***	***
Bifix SE	***	***		***	***	**	***	***	**	***	***	***	***
Core X Flow	***	***	***		***	***		***	***		***		
RelyX Unicem 2			***	***		***	***	***	***	***	***	***	*
SmartCem2	***	***	**	***	***		***		***	***	***	***	***
Calibra	***	***	***		***	***		***	**		***		
BeautiCem	***	***	***	***	***		***		***	***	***	***	***
SoloCem	***	***	**	***	***	***	**	***		***	***		***
DuoCem	***	***	***		***	***		***	***		***		
Permazem	***	***	***	***	***	***	***	***	***	***		***	***
Panavia 2.0	***	***	***		***	***		***			***		
Variolink Esthetic	***	***	***		*	***		***	***		***		

**hFOB cells**	Hoffmann's PZ	RelyX Ultimate	Bifix SE	Core X Flow	RelyX Unicem 2	SmartCem2	Calibra	BeautiCem	SoloCem	DuoCem	Permazem	Panavia 2.0	Variolink Esthetic
Hoffmann's PZ		***	***	***	***	***	***	***	***	***	***	***	***
RelyX Ultimate	***		**	**	***	**		**	**			**	***
Bifix SE	***	**		***	***								***
Core X Flow	***	**	***			***	***	***	***	***	***	***	***
RelyX Unicem 2	***	***	***			***	***	***	***	***	***	***	***
SmartCem2	***	**		***	***								***
Calibra	***			***	***								***
BeautiCem	***	**		***	***								***
SoloCem	***	**		***	***								***
DuoCem	***			***	***								***
Permazem	***			***	***								***
Panavia 2.0	***	**		***	***								***
Variolink Esthetic	***	***	***	***	***	***	***	***	***	***	***	***	

**Saos-2 cells**	Hoffmann's PZ	RelyX Ultimate	Bifix SE	Core X Flow	RelyX Unicem 2	SmartCem2	Calibra	BeautiCem	SoloCem	DuoCem	Permazem	Panavia 2.0	Variolink Esthetic
Hoffmann's PZ		***	***	***	***	***	***	***	***	***	***		***
RelyX Ultimate	***		***		*	***		***	***	***	***	***	***
Bifix SE	***	***		***	***		***		**	***	***	***	***
Core X Flow	***		***			***		***	***	***	***	***	***
RelyX Unicem 2	***	*	***			***		***	***	***	***	***	***
SmartCem2	***	***		***	***		***		***	***	**	***	***
Calibra	***		***			***		***	***	***	***	***	***
BeautiCem	***	***		***	***		***		***	***	***	***	***
SoloCem	***	***	**	***	***	***	***	***		***	***	***	***
DuoCem	***	***	***	**	***	***	***	***	***		***	***	***
Permazem	***	***	***	***	***	**	***	***	***	***		***	***
Panavia 2.0		***	***	***	***	***	***	***	***	***	***		***
Variolink Esthetic	***	***	***	***	***	***	***	***	***	***	***	***	
